# Modeling of kidney allograft rejection using hiPSC-derived kidney organoids and HLA-mismatched PBMCs: an *in vitro* co-culture system

**DOI:** 10.1007/s00018-025-05867-7

**Published:** 2025-09-02

**Authors:** Sun Woo Lim, Sheng Cui, Xianying Fang, Do Hyun Na, Hanbi Lee, Yoo Jin Shin, Hyunhye Kang, Eun-Jee Oh, Byung Ha Chung

**Affiliations:** 1 Transplantation Research Center, Seoul St. Mary’s Hospital, College of Medicine, The Catholic University of Korea, Seoul, Korea; 2 Division of Nephrology, Department of Internal Medicine, Seoul St. Mary’s Hospital, College of Medicine, The Catholic University of Korea, 222 Banpo-daero, Seocho-Gu, Seoul, 06591 South Korea; 3 Department of Laboratory Medicine, The Catholic University of Korea, Seoul St. Mary’s Hospital, Seoul, Korea

**Keywords:** Kidney organoid, Kidney transplantation, Allograft rejection, HiPSC

## Abstract

The aim of this study is to establish an *in vitro* co-culture system to model allograft rejection using kidney organoids system derived from human induced pluripotent stem cells (hiPSCs). We co-cultured kidney organoids derived from wild-type hiPSCs with HLA-mismatched peripheral blood mononuclear cells (PBMCs) from healthy controls (HC) for 24 h. To assess allogeneic rejection modeling, we measured the expression of HLA molecules, (HLA-ABC and HLA-DR), and evaluated cellular damage in the kidney organoids. Additionally, we analyzed the distribution of T cells and their subsets within the co-cultured PBMCs. The immunosuppressive effect of tacrolimus was also evaluated in this co-culture system. Transcriptomic analysis, conducted using RNA sequencing, identified molecules associated with allogeneic rejection. When kidney organoids were co-cultured with alloreactive PBMCs for 24 h, HLA-ABC and HLA-DR expression significantly increased in kidney organoid cells. Additionally, kidney organoids showed reduced cell viability and increased apoptosis compared to syngeneic controls, as assessed by flow cytometry and Annexin V/PI staining. However, treatment with tacrolimus reduced HLA expression in a dose-dependent manner, highlighting the diminished alloimmune responses. Further analysis of PBMC subsets revealed shifts in T helper (TH) and cytotoxic T cell (TC) populations under allogeneic conditions, including increased effector TH and TC cells. Transcriptomic analysis through RNA sequencing identified 256 differentially expressed genes (DEGs), with notable immune-related pathways such as NF-kappa B and TNF signaling involved in allograft rejection. These results provide evidence that a co-culture system with allogeneic kidney organoids and PBMCs can potentially model transplant rejection in vitro.

**Supplementary Information:**

The online version contains supplementary material available at 10.1007/s00018-025-05867-7.

## Introduction

Kidney transplantation is the most effective treatment for end-stage renal disease, offering patients significantly improved quality of life and longevity [1, 2]. However, despite advancements in transplantation operation techniques and immunosuppressive therapies, allograft rejection continues to be a major challenge, adversely affecting both short- and long-term outcomes [3–5]. The complex interaction between the donor kidney and the recipient’s immune system ultimately determines the transplant’s fate, with allo-immune responses playing a pivotal role in graft recognition and subsequent rejection [6]. Thus, it is essential to employ suitable transplant rejection models to elucidate the intricate mechanisms of allograft rejection and to further research in developing effective, stable immunosuppressive agents.

Similar to other kidney diseases, animal or *in vitro* cell models have been employed in transplant rejection research [7]. However, these models of transplant rejection exhibit notable limitations. Particularly, unlike other animal models for kidney diseases, the construction of a transplantation model necessitates advanced surgical skills [8]. Consequently, large-scale animal allograft rejection model research demands substantial resources, including financial, time, and specialized human resources [9]. Moreover, significant differences in immune responses and metabolic pathways necessitate careful interpretation to accurately predict outcomes in humans [9]. These factors may result in smaller-scale studies or interruptions in research due to resource limitations. In this context, *in vitro* models that accurately reflect the complex dynamics of allograft rejection are indispensable for uncovering the underlying mechanisms and for developing innovative therapeutic strategies. However, traditional monolayer cell cultures are often inadequate in representing the three-dimensional architecture and cellular interactions typical of the *in vivo* environment [10, 11]. Therefore, the development of more physiologically pertinent models that can accurately simulate the interactions between donor tissue and recipient immune cells is urgently needed.

Meanwhile, over the past decade, kidney organoids derived from human induced pluripotent stem cells (hiPSCs) have emerged as pioneering *in vitro* models for kidney disease research [12–15]. Compared to traditional animal or *in vitro* 2D culture cell models, kidney organoid systems offer several advantages for kidney disease research, presenting a more effective avenue for studying complex kidney conditions. For example, employing this system can overcome the limitations of animal models, which often diverge from human tissues; moreover, it facilitates the creation of sophisticated 3D human kidney tissues that are not achievable with 2D cell cultures [16–18]. Until now, the kidney organoid system has successfully modeled various kidney diseases such as autosomal dominant polycystic kidney disease and Fabry disease nephropathy [19–22]. However, *in vitro* modeling of kidney allograft rejection using a hiPSC -derived kidney organoid system has yet to be attempted.

To address this need, we present a robust *in vitro* model designed to investigate kidney transplant rejection. This model utilizes three-dimensional co-culture systems that incorporate kidney organoids derived from hiPSCs and alloreactive peripheral blood mononuclear cells (PBMCs). This model provides a platform for detailed studies on the pathophysiology of allograft rejection within a controlled laboratory environment.

## Materials and methods

### Peripheral blood mononuclear cell isolation

PBMCs were harvested via sterile venipuncture, collected into citrate tubes (ACD; Becton–Dickinson, Franklin Lakes, NJ, USA), and subsequently purified using Ficoll-density centrifugation as per the manufacturer’s protocol (Cellgro, Mediatech, Inc., Herndon, VA, USA). Following purification, the cell count and viability of PBMCs were determined using an automated cell counter (ADAM MC; NanoEntek Inc., Hwaseong-si, Gyeonggi-do, South Korea). PBMC were resuspended in 4 °C cryomedia (CryoSTOR® CS10 #100–1061; STEMCELL Vancouver, Canada) at a concentration of 1 × 10^6^ cells/ml, and aliquoted in 1 ml per cryotube (43021; SPL Life Sciences, Pocheon-si, Gyeonggi-do, South Korea). The study was approved by Institutional Review Board of Seoul St. Mary’s Hospital (KC19TISI0901). All subjects provided informed consent for inclusion before they participated in this study. This study was conducted in accordance with the Declaration of Helsinki.

### Kidney organoids differentiation from hiPSCs and co-culture with PBMC

Healthy control (HC) hiPSCs were generated from PBMCs of a healthy individual and validation process of successful reprogramming into hiPSCs is presented in supplemental method and Fig. S1. Wild-type (WT) hiPSCs (WTC-11) and HC hiPSCs were differentiated into kidney organoids following the previously published protocol [13, 19, 23]. Initially, hiPSCs were plated in mTeSR1 medium (05850; STEMCELL Technologies, Vancouver, Canada) supplemented with 10 μM Y-27632 (1293823; Biogems, Westlake Village, CA, USA) onto 24-well plates pre-coated with 1.25% Corning Matrigel® hESC Qualified Matrix. After 24 h, the medium was replaced with mTeSR1 containing 2.5% Matrigel®. On the fourth day, the medium was switched to Advanced RPMI (1263302; Thermo Fisher Scientific, Grand Island, NY, USA) supplemented with 12 µM CHIR-99021 (STEMCELL Technologies). Approximately 36 h later, the medium was exchanged for Advanced RPMI enhanced with B27 supplement (17504044, Thermo Fisher Scientific). Organoids continued to be cultured in this medium until they were collected on day 21. Regarding the co-culture, kidney organoids from either WT or HC hiPSCs were co-cultured with PBMCs (1 × 10^6^ cells/1.9 cm^2^ in 500 ul RB media) from HC hiPSCs on day 20 of differentiation for 24 h. The following day, both PBMCs and kidney organoids were harvested for further experiments. To ensure reproducibility, all experiments were performed in triplicate.

### Human leucocyte antigen (HLA) genotyping and haplotype match

HLA typing was conducted at the Histocompatibility Laboratory at Seoul ST Mary’s Hospital. WT and HC hiPSCs were analyzed for HLA-A*, -B*, -C*, -DRB1*, and –DQB1* loci using high -resolution next-generation sequencing (NGS) and the specific mismatches at each loci are detailed in Table S1. Libraries were prepared according to the manufacturer’s protocol using the AllType FAST plex NGS 11 Loci Kit (One Lambda, West Hills, CA, USA) and run on the Ion Torrent S5 XL platform (Thermo Fisher Scientific, Waltham, MA, USA). The resulting reads were analyzed with TypeStream Visual software (One Lambda) to establish HLA genotypes based on the IPD-IMGT/HLA database version 3.51.

Haplotype match or HLA-identity in WT-hiPSC and HC hiPSC was evaluated based on HLA -A*, -B*, -C*, -DRB1*, and –DQB1* typing following standard criteria. The HLA-ID group primarily comprised sibling transplants, whereas the 1 haplotype-mismatched group included both sibling transplants and parent–child transplants. HLA-DR match in cadaveric RTx was analyzed by interpreting ‘blanks’ as indicators of homozygosity and by counting each allele as a separate antigen. For instance, a homozygous DR 1, – recipient of a homozygous DR 1, – RTx was identified as a 2 DR match; a homozygous DR 1, – recipient of a homozygous DR 2, – donor RTx was categorized as a 0 DR match; a homozygous DR 1, – recipient of a RTx from a heterozygous donor (DR 1, 2) was acknowledged as a 1 DR match, etc.

### Flow cytometry

hiPSCs cells were dissociated using TE (15400054; Life Technologies). Subsequent to being washed twice with FACS buffer (phosphate-buffered saline [PBS] containing 1% bovine serum albumin and 10 mM sodium azide), permeabilized for 30 min using flow cytometry fixation and permeabilization solution (554714; BD Biosciences, San Jose, CA, USA), washed with wash buffer, and stained with antibodies listed in the Table S2. Cells were analyzed using a FACS Canto II flow cytometer (BD Biosciences). The data were analyzed using FlowJo™ Software v10.10 (Becton, Dickinson & Company, Ashland, OR, USA).

### Immunofluorescence

Kidney organoids were washed once with phosphate-buffered saline (PBS), fixed in 4% paraformaldehyde for 10 min at 4 °C, and blocked in 5% donkey serum in PBS-T (0.3% Triton X-100 in PBS) for 1 h at room temperature (RT). Following this, kidney organoids were incubated with the primary antibodies listed in Table S3. Nucleic acid staining was conducted by incubating with 4′,6-diamidine-2-phenylindole (10236276001, DAPI, 1:5000; Roche, Basel, Switzerland) for 30 min at room temperature. Images were captured using a Zeiss LSM700 confocal microscope (Carl Zeiss MicroImaging GmbH, Jena, Germany).

### Annexin V and PI assay

Harvested kidney organoids on day 21 after differentiation were dissociated using TE (15400054; Life Technologies), then were incubated with 5 μl of fluorescein isothiocyanate-conjugated annexin V (BD Biosciences) and 2 μl of PI (Propidium Iodide) in 1 × binding buffer (BD Biosciences) for 15 min at RT, in accordance with the manufacturer’s protocol. The stained cells were subjected to flow cytometry analysis using a FACS Canto II instrument. The results are presented as the percentage of fluorescent cells relative to the total cell count.

### qRT-PCR

Total RNA was extracted from cells using RNA-Bee (CD-105B; Tel-Test, Friendswood, TX, USA) following the manufacturer’s instructions. First-strand cDNA using RNA was synthesized and placed onto quantitative real-time PCR (qRT-PCR) using cDNA systhesis kit (DYRT1120; Dyne Bio Inc, Seongnamsi, South Korea). Relative gene level was normalized to GAPDH level using the change-in-threshold method. Primer sequences are shown in Table S4.

### Library preparation and sequencing

For control and test RNAs, library construction was performed using the QuantSeq 3’ mRNA-Seq Library Prep Kit (Lexogen, Inc., Austria), following the manufacturer’s instructions. Specifically, total RNA samples (500 ng each) were prepared; an oligo-dT primer with an Illumina-compatible sequence at its 5′ end was hybridized to the RNA and reverse transcribed. Post reverse transcription, the RNA template was degraded, and second-strand synthesis was initiated using a random primer that also contained an Illumina-compatible linker sequence at its 5′ end. Subsequently, the double-stranded library was purified using magnetic beads to eliminate all reaction components. The library then underwent amplification to incorporate the full adapter sequences necessary for cluster generation. The final library was purified to remove PCR components. High-throughput sequencing was conducted as single-end 75-bp sequencing on a NextSeq 500 system (Illumina, Inc., USA).

### Data analysis

QuantSeq 3 mRNA-Seq reads were aligned using Bowtie2 [24]. Bowtie2 indices were generated from either the genome assembly sequence or representative transcript sequences for alignment to both the genome and transcriptome. The alignment files were utilized for transcript assembly, estimating abundance, and identifying differential gene expression. Differentially expressed genes (DEGs) were identified based on counts from both unique and multiple alignments using coverage analysis in BEDtools [25]. The read count (RC) data were analyzed using the TMM + CPM normalization method with EdgeR within R (R development Core Team, 2020) on Bioconductor [26]. Gene classification was conducted using searches performed in the DAVID (http://david.abcc.ncifcrf.gov/) and Medline databases (https://www.ncbi.nlm.nih.gov/).

### Statistical analyses

Data are presented as the mean ± standard error (SE) of at least three independent experiments. Multiple comparisons between groups were conducted using a one-way analysis of variance with a Bonferroni *post hoc* test, employing Prism software (version 10.2.0 for Windows; GraphPad Software, La Jolla, CA, USA). Statistical significance was established at *P* < 0.05.

## Results

### Three-dimensional co-culture system using kidney organoids and alloreactive PBMC for inducing allogeneic condition

**Fig. 1:**
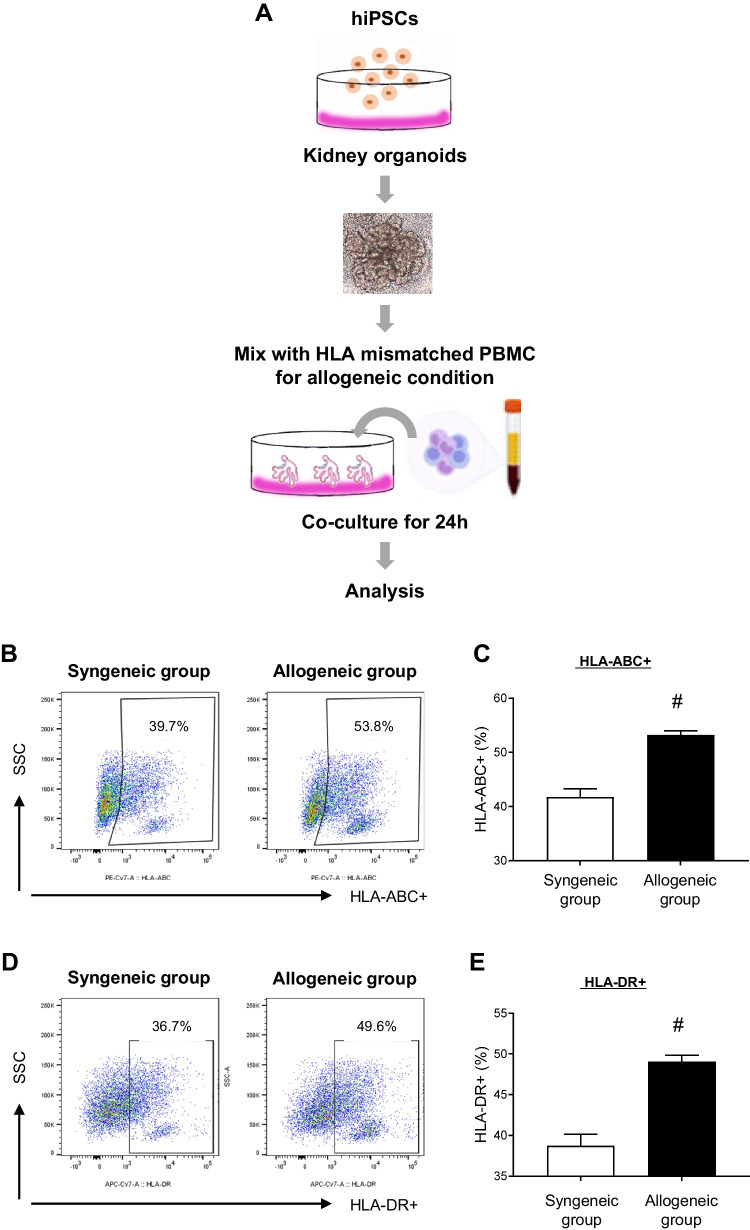
Vitro recapitulation of allogeneic conditions using co-culture with HLA-mismatched PBMCs and kidney organoids. (**A**) Schematic illustration of experimental conditions for allogeneic conditions using HLA-mismatched PBMCs and kidney organoids derived from hiPSCs. Representative flow cytometric dot plots and graphs showing the HLA-ABC + cells (**B** and **C**) and HLA-DR + cells (**D** and **E**) of kidney organoids in the syngeneic group and allogeneic group. Data is presented as mean ± SE. #*P* < 0.05 vs. syngeneic group

To set up our in vitro kidney transplant rejection model, which we refer to as the allogeneic condition in a culture dish, we initially conducted HLA mismatch scoring for HLA-A, B, C, DRB1, and DQB1 loci in hiPSCs employed in this study. Table S1 presents the results of high-resolution WT-hiPSC and HC PBMC HLA-typing for both class-I (A, B, C) and class-II (DRB1, DQB1) antigens using NGS technology. We established a co-culture system by combining a kidney organoid derived from WT-hiPSC with HLA-mismatched PBMC from HC for 24 h as described in the experimental workflows (Fig. 1A). For the syngeneic control group, we established an HC hiPSC line from this HC’s PBMC using a reprogramming method with episomal vectors (Fig. S1). Primary humoral alloimmune activation following allogeneic conditions in the co-culture system was assessed by quantifying the induction of HLA molecules, HLA-ABC, and -DR in whole-well cells via flow cytometric analysis. The proportion of HLA-ABC and HLA-DR-positive cells significantly increased in the allogeneic group compared to the syngeneic group (HLA-ABC, 53.2 ± 0.4% vs. 41.8 ± 0.7%, ^*#*^*P* < 0.05 vs. syngeneic group; HLA-DR, 38.8 ± 0.7% vs. 49.1 ± 0.4%, ^*#*^*P* < 0.05 vs. syngeneic group) (Figs. 1B-E).

### Increased HLA expression in the kidney organoids in the allogeneic condition

**Fig. 2:**
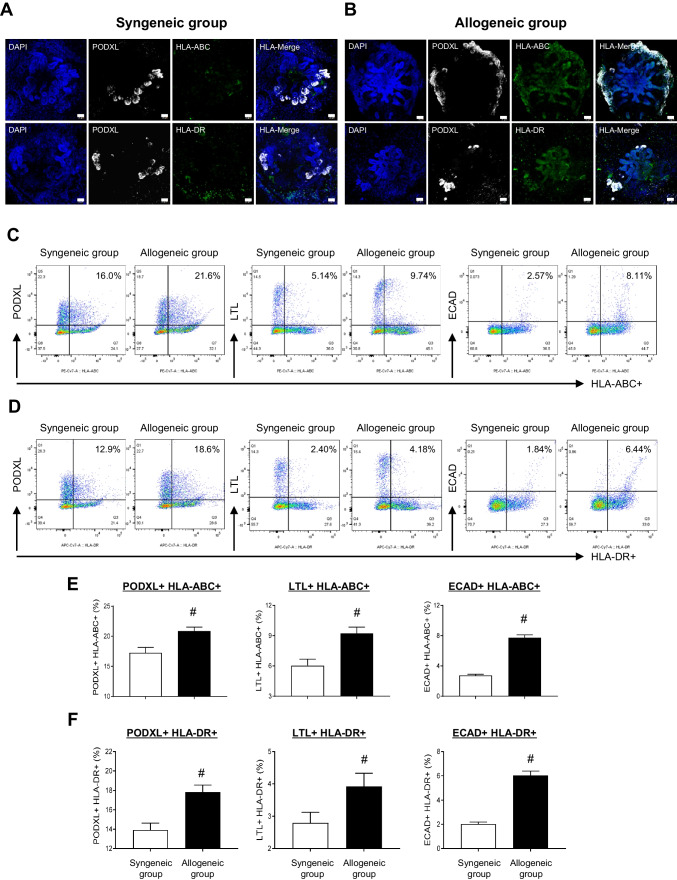
Expression of HLA-ABC and HLA-DR in the kidney organoids after co-culture with HLA-mismatched PBMC. After co-culturing with PBMC and maturing kidney organoids for 24 h, the kidney organoids were double labeled with antibodies against HLA-ABC or HLA-DR, as well as PODXL, under both the syngeneic condition (**A**) and the allogeneic condition (**B**). Scale bar = 50 μm. Representative flow cytometric dot plots and their quantitative graphs show the HLA-ABC + cells (**C** and **E**) and HLA-DR + cells (**D** and **F**) in the nephron markers, PODXL, LTL, and ECAD in the syngeneic group and allogeneic group. PODXL, podocalyxin; LTL, lotus tetragonolobus lectin; ECAD, e-cadherin. Data are presented as mean ± SE. ^#^*P* < 0.05 vs. syngeneic group

Next, we assessed the expression of HLA molecules in the nephron structure, including podocalyxin (PODXL) in glomerular epithelial cells, lotus tetragonolobus lectin (LTL) in the proximal tubules, and e-cadherin (ECAD) in the distal tubules. Immunofluorescence staining and examination with confocal microscopy revealed that immunoreactivity for HLA-ABC and –DR was elevated in the allogeneic group compared to the syngeneic group (Fig. 2A and B). Following co-culture with alloreactive PBMC, the kidney organoids disintegrated into single cells. These cells were then stained with a cocktail of antibodies targeting HLA-ABC, -DR, PODXL, LTL, and ECAD for flow cytometric analysis. Flow cytometric dot plots and quantitative graphs demonstrated that the percentages of HLA-ABC, -DR-positive cells containing nephron structure markers were significantly higher in the allogeneic group than in the syngeneic group (PODXL + HLA-ABC +, 20.9 ± 0.4% vs. 17.3 ± 0.5%, ^*#*^*P* < 0.05 vs. syngeneic group; LTL + HLA-ABC +, 9.2 ± 0.4% vs. 6.0 ± 0.3%, ^*#*^*P* < 0.05 vs. syngeneic group; ECAD + HLA-ABC +, 7.7 ± 0.2% vs. 2.8 ± 0.1%, ^*#*^*P* < 0.05 vs. syngeneic group; PODXL + HLA-DR +, 17.8 ± 0.4% vs. 13.9 ± 0.4%, ^*#*^*P* < 0.05 vs. syngeneic group; LTL + HLA-DR +, 3.9 ± 0.2 vs. 2.8 ± 0.2%, ^*#*^*P* < 0.05 vs. syngeneic group; ECAD + HLA-DR +, 6.0 ± 0.2% vs. 2.0 ± 0.1%, ^*#*^*P* < 0.05 vs. syngeneic group) (Fig. 2C-F).

### Reduced cell viability of the kidney organoids in the allogeneic condition

**Fig. 3:**
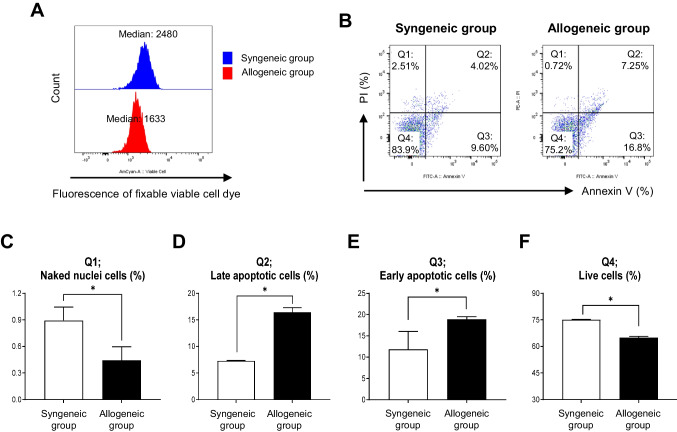
Effect of cell viability of the kidney organoids after co-culture with HLA-mismatched PBMC and kidney organoids. (**A**) Representative histogram showing cell viability measured by a fixable viable cell dye in syngeneic and allogeneic groups of kidney organoids. (**B**) Flow cytometry analysis of apoptotic cell death induced under allogeneic conditions using annexin V-FITC/PI staining. (**C**-**F**) The gating strategy includes single PI positive (Q1), double positive (Q2), single annexin V positive (Q3), and double negative (Q4). Data are presented as mean ± SE. *P < 0.05 vs. syngeneic group

We investigated the effects of HLA-mismatched PBMC on the viability of kidney organoid cells in a co-culture system. The viable cells were labeled using a fluorescence fixable viable cell dye. A histogram of cell viability displayed a reduced median value in the allogeneic group at 1633 compared with 2480 in the syngeneic group (Fig. 3A). The Annexin V and PI assay indicated that early (Annexin V positive, PI negative) and late apoptotic cells (Annexin V and PI positive) were significantly more prevalent in the allogeneic group than in the syngeneic group (Q2, 16.4 ± 0.5% vs. 7.3 ± 0.03%, *P < 0.05 vs. the syngeneic group; Q3, 18.9 ± 0.4% vs. 11.9 ± 2.4%, *P < 0.05 vs. the syngeneic group). Consistent with the result of Fig. 3A, the percentage of live cells (Annexin V and PI negative) was also lower in the allogeneic group compared to the syngeneic group (65.0 ± 0.4% vs. 75.1 ± 0.1%, *P < 0.05 vs. the syngeneic group).

### Tacrolimus reduced the HLA expression in a dose -dependent manner

**Fig. 4:**
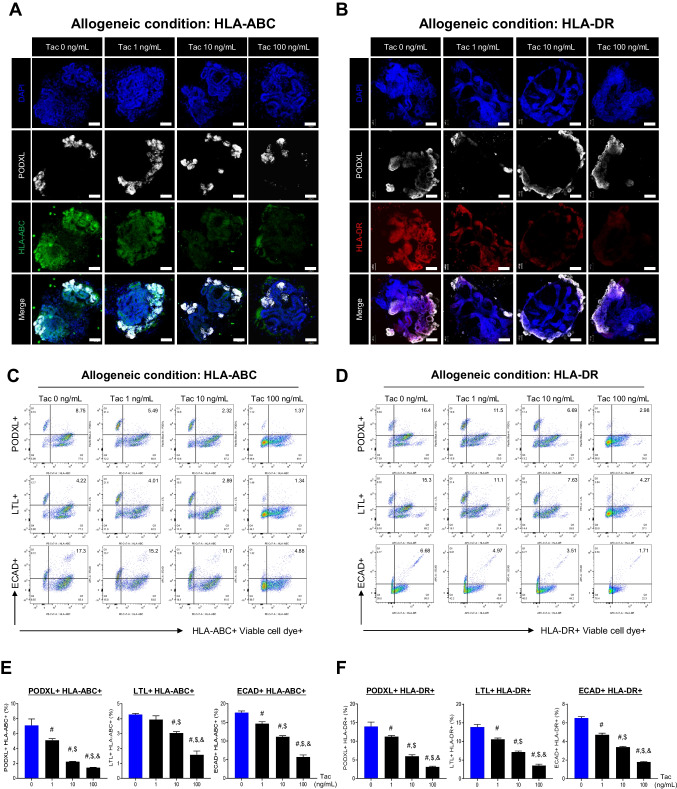
Effect of tacrolimus on the expression of HLA-ABC and HLA-DR in the kidney organoids after co-culture with HLA-mismatched PBMC. Tacrolimus was administered during the co-culture of PBMC with mature kidney organoids for 24 h. Subsequently, the kidney organoids were double -labeled with antibodies against HLA-ABC (**A**) or HLA-DR (**B**) and PODXL under an allogeneic condition. Scale bars represent 50 μm. Representative flow cytometric dot plots and corresponding quantitative graphs illustrate HLA-ABC + cells (**C** and **E**) and HLA-DR + cells (**D** and **F**) among the nephron markers PODXL, LTL, and ECAD in the allogeneic group. Tac, tacrolimus; PODXL, podocalyxin; LTL, lotus tetragonolobus lectin; ECAD, e-cadherin. Data are presented as mean ± SE. ^#^*P* < 0.05 vs. 0 ng/mL of Tac group; ^$^*P* < 0.05 vs. 1 ng/mL of Tac group; ^&^*P* < 0.05 vs. 10 ng/mL of Tac group

Figure 4 illustrates the impact of tacrolimus (Tac) treatment on HLA-ABC and -DR expression in kidney organoids under allogeneic conditions. Immunofluorescence staining and confocal microscopy revealed a gradual decrease in the immunoreactivity of HLA-ABC and -DR as the Tac dose was increased (Fig. 4A and B). Additionally, flow cytometric dot plots and their quantitative graphs also indicate that Tac treatment significantly reduced the percentage of HLA-ABC and -DR-positive cells across all nephron structure markers - PODXL, LTL, and ECAD- in a Tac dose -dependent manner relative to the group treated with 0 ng/mL of Tac (PODXL + HLA-ABC +, 7.1 ± 0.9% in the Tac 0 ng/mL, 5.1 ± 0.2% in the Tac 1 ng/mL, 2.2 ± 0.04% in the Tac 10 ng/mL, 1.4 ± 0.04% in the Tac 100 ng/mL; LTL + HLA-ABC +, 4.3 ± 0.1% in the Tac 0 ng/mL, 4.0 ± 0.2% in the Tac 1 ng/mL, 3.0 ± 0.1% in the Tac 10 ng/mL, 1.6 ± 0.2% in the Tac 100 ng/mL; ECAD + HLA-ABC +, 17.7 ± 0.4% in the Tac 0 ng/mL, 14.7 ± 0.5% in the Tac 1 ng/mL, 11.1 ± 0.3% in the Tac 10 ng/mL, 5.8 ± 0.5% in the Tac 100 ng/mL; PODXL + HLA-DR +, 14.0 ± 1.2% in the Tac 0 ng/mL, 11.2 ± 0.3% in the Tac 1 ng/mL, 6.0 ± 0.4% in the Tac 10 ng/mL, 3.2 ± 0.2% in the Tac 100 ng/mL; LTL + HLA-DR +, 13.9 ± 0.7% in the Tac 0 ng/mL, 10.6 ± 0.3% in the Tac 1 ng/mL, 7.2 ± 0.2% in the Tac 10 ng/mL, 3.5 ± 0.4% in the Tac 100 ng/mL; ECAD + HLA-DR +, 6.5 ± 0.1% in the Tac 0 ng/mL, 4.7 ± 0.2% in the Tac 1 ng/mL, 3.4 ± 0.1% at Tac 10 ng/mL, 1.8 ± 0.05% in the Tac 100 ng/mL, ^#^*P* < 0.05 vs. 0 ng/mL of Tac group; ^$^*P* < 0.05 vs. 1 ng/mL of Tac group; ^&^*P* < 0.05 vs. 10 ng/mL of Tac group).

### Influence on helper and cytotoxic T cell subsets of the PBMC under in vitro allogeneic condition

**Fig. 5:**
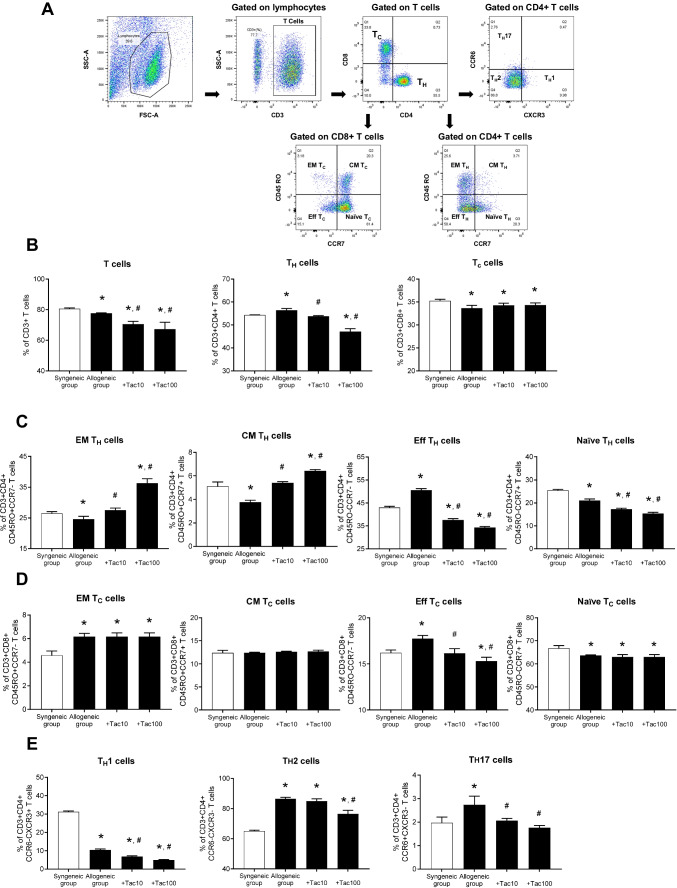
Comparisons of CD3 + T cell subsets of PBMC after co-culturing with HLA-mismatched kidney organoids between the syngeneic and allogeneic groups with or without Tac. (**A**) Gating strategy for T cell subsets. (**B**) Percentage of total T (CD3 +), T_H_ (CD3 + CD4 +), and T_C_ (CD3 + CD8 +) cells in each group. (**C**) Percentage of T_H_ subsets, EM T_H_ (CD3 + CD4 + CD45RO + CCR7-), CM T_H_ (CD3 + CD4 + CD45RO + CCR7 +), Eff TH (CD3 + CD4 + CD45RO- CCR7-), and Naïve TH cells (CD3 + CD4 + CD45RO- CCR7 +) in each group. (**D**) Percentage of T_C_ subsets, EM TC (CD3 + CD8 + CD45RO + CCR7-), CM T_C_ (CD3 + CD8 + CD45RO + CCR7 +), Eff T_C_ (CD3 + CD8 + CD45RO- CCR7-), and Naïve T_C_ (CD3 + CD8 + CD45RO- CCR7 +) cells. (**E**) Percentage of T_H_ subsets, T_H_1 (CD3 + CD4 + CCR6- CXCR3 +), T_H_2 (CD3 + CD4 + CCR6- CXCR3-), and T_H_17 (CD3 + CD4 + CCR6 + CXCR3-) cells. T_H_, Helper T cells; T_C_ cytotoxic T cells; EM, effector memory; CM, central memory; Eff, effector; PBMC, Peripheral blood mononuclear cell; Tac, Tacrolimus Data are presented as mean ± SE. *P < 0.05 vs. syngeneic group, ^#^*P* < 0.05 vs. allogeneic group without tacrolimus

In this study, we analyzed the distribution of T cells (CD3^+^) and their subsets, i.e., T_H_ (CD4^+^) and T_C_ (CD8^+^) cells. Based on CD45RO and CCR7 surface expression levels, both T_H_ and T_C_ cells were further classified into four subsets: naïve (CD4^+^CD45RO^-^CCR7^+^), central memory (CM; CD4^+^CD45RO^+^CCR7^+^), effector memory (EM; CD4^+^CD45RO^+^CCR7^-^), and effector (CD4^+^CD45RO^-^CCR7^+^) cells. Additional subsets of T_H_ cells analyzed included T_H_1 (CD4^+^CXCR3^+^CCR6^-^), T_H_2 (CD4^+^CXCR3^-^CCR6^-^), and T_H_17 (CD4^+^CXCR3^-^CCR6^+^). The gating strategies are depicted in Fig. 5A.

To examine the immunosuppressive effect of tacrolimus, PBMCs were co-cultured with mature kidney organoids under allogeneic conditions for 24 h in the presence or absence of tacrolimus (10 or 100 ng/mL). In Fig. 5B, the frequencies of total T cells, T_H_ cells, and T_C_ cells were significantly altered in the allogeneic group compared to the syngeneic group, (T cells, 78 ± 0.2% vs. 81 ± 0.3%; T_H_ cells, 56 ± 0.4% vs. 55 ± 0.2%; T_C_ cells, 34 ± 0.4% vs. 35 ± 0.2%, *P < 0.05 vs. syngeneic group) and these changes were attenuated by tacrolimus treatment (T cells, 67 ± 3.2% and 71 ± 1.3%; T_H_ cells, 54 ± 0.2% and 47 ± 1.2%; T_C_ cells, 34 ± 0.3% and 34 ± 0.3%, *P < 0.05 vs. syngeneic group, ^*#*^*P* < 0.05 vs. allogeneic group without tacrolimus, allogeneic group + Tac10 or Tac100, respectively). As shown in Fig. 5C, the percentage of effector T_H_ cells was significantly elevated in the allogeneic group (51 ± 0.4% vs. 43 ± 0.3%, *P < 0.05 vs. syngeneic group), while EM and CM T_H_ cells were reduced (EM T_H_ cells, 25 ± 0.6% vs. 27 ± 0.3%; CM T_H_ cells, 3.8 ± 0.1% vs. 5.1 ± 0.2%, *P < 0.05 vs. syngeneic group); these changes were partially reversed by tacrolimus treatment (Eff T_H_ cells, 38 ± 0.4% and 34 ± 0.3%; EM T_H_ cells, 28 ± 0.5% and 37 ± 0.9%; CM T_H_ cells, 5.4 ± 0.1% and 6.4 ± 0.1%, *P < 0.05 vs. syngeneic group, ^*#*^*P* < 0.05 vs. allogeneic group without tacrolimus, allogeneic group + Tac10 or Tac100, respectively). Similarly, effector T_C_ cells and EM T_C_ cells were significantly increased in the allogeneic group (EM T_C_ cells, 6.2 ± 0.2% vs. 4.6 ± 0.2%; Eff T_C_ cells, 17.7 ± 0.2% vs. 16.2 ± 0.2%, *P < 0.05 vs. syngeneic group), and tacrolimus treatment significantly suppressed the increase in effector T_C_ cells (Eff T_C_ cells, 16 ± 0.4% and 15 ± 0.3%, *P < 0.05 vs. syngeneic group, ^*#*^*P* < 0.05 vs. allogeneic group without tacrolimus, allogeneic group + Tac10 or Tac100, respectively) (Fig. 5D). Furthermore, as shown in Fig. 5E, the proportion of T_H_1 cells was markedly decreased in the allogeneic group and further reduced by tacrolimus treatment. (T_H_1 cells, 10.5 ± 0.2% vs. 31.2 ± 0.3%; *P < 0.05 vs. syngeneic group; 6.9 ± 0.2% and 5 ± 0.3%. ^*#*^*P* < 0.05 vs. allogeneic group without tacrolimus, allogeneic group + Tac10 or Tac100, respectively). In contrast, the proportion of T_H_2 and T_H_17 cells was elevated in the allogeneic group compared to the syngeneic group (T_H_2 cells, 86.5 ± 0.6% vs. 65.2 ± 0.3%; T_H_17 cells, 2.7 ± 0.1% vs. 2.0 ± 0.1%, *P < 0.05 vs. syngeneic group), and this increase was partially suppressed by tacrolimus at both concentrations (T_H_2 cells, 85 ± 1.1% and 77 ± 1.6%; T_H_17 cells, 2.1 ± 0.1% and 1.8 ± 0.1%, ^*#*^*P* < 0.05 vs. allogeneic group without tacrolimus, allogeneic group + Tac10 or Tac100, respectively).

### Transcriptomic analysis of the kidney organoids derived allogeneic condition versus syngeneic condition

**Fig. 6:**
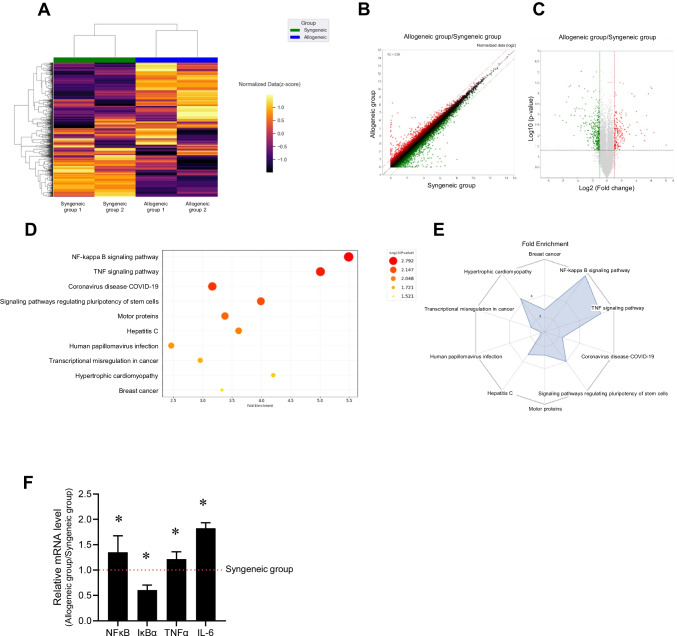
Whole transcriptome analysis of the kidney organoids of allogeneic group versus syngeneic group. (**A**) Heat map and hierarchical clustering of RNA-sequencing data from the kidney organoids of both the syngeneic and allogeneic groups. Scatter plot (**B**) and volcano plot (**C**) illustrate the significant transcriptional changes in genes associated with allogeneic conditions. (**D**) The size and color of each bubble indicate the number of significantly changed genes enriched in the pathway and –log10 (p-value), respectively. The Y-axis represents the pathway name, and the x-axis represents the fold enrichment factor. (**E**) A radar chart displays normalized enrichment scores for genes with significant transcriptional changes in the allogeneic group across hallmark pathways; pathways more overrepresented in the allogeneic group than in the syngeneic group are highlighted with red brackets. (**F**) qRT-PCR result for mRNA level of NF-κB, IκBα, TNFα, and IL-6 in the kidney organoids. Data are presented as mean ± SE. *P < 0.05 vs. syngeneic group

To investigate the differences in gene expression profiles of kidney organoids between syngeneic and allogeneic conditions, we conducted a transcriptomic analysis using RNA-sequencing. The results are illustrated in the hierarchical clustering heat map (Fig. 6A), scatter plot (Fig. 6B), and volcano plot (Fig. 6C), which depict global transcriptomic variations between the groups. A total of 43,424 RNA transcripts were identified in the kidney organoids; moreover, we discovered 256 DEGs with a *p*-value of < 0.05. Among these, 93 genes were two-fold upregulated while 163 genes were two-fold downregulated. Additionally, a bubble or radar chart displays the Kyoto Encyclopedia of Genes and Genomes (KEGG) enrichment analysis, predicting the potential functions of the DEGs (Fig. 6D and E). The NF-κB and TNFα signaling pathways were ranked at the top, suggest that the co-cultivation of kidney organoids with HLA-mismatched PBMCs reflects kidney allograft rejection. Figure 6 presents the qRT-PCR results for NF-κB, IκBα, TNFα, and IL-6 which play critical roles in the NF-κB and TNFα signaling pathways (NF-κB, 1.35 ± 0.32; IκBα, 0.60 ± 0.1; TNFα, 1.22 ± 0.15; IL-6, 1.83 ± 0.11, *P < 0.05 vs. syngeneic group).

## Discussion

The study presented delineates a robust in vitro model for investigating kidney transplant rejection, utilizing a three-dimensional co-culture system that integrates kidney organoids derived from hiPSCs with alloreactive PBMCs. Through meticulous experimental workflows and comprehensive analyses, this study elucidates the dynamics of allogeneic conditions, highlighting critical aspects such as HLA expression, cell viability, immune cell subsets, and transcriptomic alterations.

Our first aim was to determine whether kidney organoids could be utilized in an allograft rejection model by inducing an immune response. HLA is a crucial component of the human immune system, and increased HLA expression on kidney allograft cells can serve as a marker of immune recognition by the recipient’s immune system, potentially leading to allograft rejection [27–29]. As a preliminary step, we exposed the kidney organoids to the inflammatory cytokine IFN-γ, and observing an increase in HLA expression on the surface of kidney organoid constituent cells (Figs. S2 and 3). Subsequently, we assessed whether there was an increase in HLA expression on the surface of the organoid cells to determine if an immune response occurred between the immune cells of PBMC and kidney organoids. The human immune system perceives mismatched HLA as"foreign"and mounts an attack, which primarily manifests as phenomena such as allograft rejection [6, 27]. The more mismatched HLA there are, the stronger the immune reaction can be [30]. Therefore, we selected PBMCs and hiPSCs with completely mismatched HLA-class I and II genotyping to develop a co-culture system of PBMC- and hiPSC -derived kidney organoids. As a result, we observed heightened expression of HLA molecules, notably HLA-ABC and HLA-DR, in kidney organoids under allogeneic conditions. Furthermore, the augmentation of HLA expression across different cells constituting nephron structures, such as podocyte, proximal, or distal tubule cells, underscores the pervasive impact of alloreactive PBMCs on kidney organoid physiology.

Next, we investigated whether elevated HLA expression contributed to cell damage, as observed in the real process of allograft rejection. The upregulation of HLA expression on cells is often associated with various physiological and pathological processes, including immune activation, inflammation, and tissue injury [31, 32]. Inflammatory cytokines or chemokines released in response to HLA-mediated immune activation can recruit immune cells to the site of injury, exacerbating tissue damage through various mechanisms [33, 34]. In this study, we also found that concomitant with the upregulation of HLA expression, there was a notable reduction in cell viability within the allogeneic milieu. RNA-seq analysis also revealed that apoptosis-related gene expression profiles showed distinct clustering between allogeneic and syngeneic groups, with pro-apoptotic genes (e.g., XAF1, FAS, PMAIP1) upregulated and survival-related genes (e.g., CD24, RIPK1, PPP1R13L) downregulated in the allogeneic group (Fig. S1). This decline in cellular viability, coupled with the increased incidence of apoptotic phenomena, highlights the cytotoxic effects exerted by alloreactive PBMCs on kidney organoids. Such observations indicate the harmful effects of allogeneic interactions on the structural integrity and viability of renal tissues.

Furthermore, we have elucidated the immunomodulatory impacts of tacrolimus, an immunosuppressant, on HLA expression and cell viability within the allogeneic context. Tacrolimus exerts its immunosuppressive effect primarily by inhibiting the calcineurin pathway, thereby reducing IL-2 production and T cell activation. This suppression affects the differentiation and proliferation of both CD4^+^ helper T cells and CD8^+^ cytotoxic T cells, contributing to reduced alloimmune responses [35, 36]. In addition, Tacrolimus may indirectly influence antigen presentation through downregulation of HLA expression on antigen-presenting cells or target organ cells [6]. Consequently, tacrolimus diminishes HLA expression on the surface of allograft kidney cells, thereby reducing their immunogenicity and decreasing the likelihood of immune-mediated rejection. This agent might also reduce cell death associated with increased HLA expression [37, 38]. Notably, Tacrolimus treatment exhibits a dose-dependent reduction in HLA expression while enhancing cell viability, thereby supporting its role in attenuating alloimmune responses and maintaining graft integrity in kidney transplantation, as demonstrated on our kidney organoid model.

Additionally, we detailed the distribution of various T cell subsets within the allogeneic environment. In scenarios of HLA mismatch between kidney and immune cells in a co-culture setting, immune cell activation might occur via established pathways such as allo-recognition, direct or indirect presentation of allo-antigen, and cytokine release [6, 39, 40]. This interaction between activated immune cells and kidney cells can provoke immune-mediated damage to kidney cells, leading to conditions such as allograft rejection [27]. This study observed an increase in effector T cells and a decrease in naïve T cells, consistent with previous findings that graft infiltration by effector T cells can elevated inflammatory cytokine activity, and induce kidney transplant rejection [10, 41]. In addition, our findings demonstrated a shift in T helper cell polarization characterized by a relative decrease in Th1 cells and an increase in Th2 and Th17 cells. While Th1 cells are traditionally associated with acute cellular rejection, Th2 responses may contribute to antibody-mediated rejection and chronic graft injury through B cell activation and IgG1/IgE production [42]. Th17 cells also have been implicated in both acute and chronic allograft injury by promoting inflammation and fibrosis via IL-17–mediated pathways. These observations are consistent with previous reports showing that Th17 cell infiltration correlates with the severity of acute T cell–mediated rejection in renal biopsies and that IL-17 neutralization can prolong graft survival in animal models [41, 43, 44]. Thus, the noteworthy changes in T cell subsets observed in PBMCs in our in vitro rejection model likely mirrors the graft damage associated with common clinical graft rejection scenarios.

Lastly, we explored whether variations in the co-culture system, such as increased HLA expression and the activation of immune cells, corresponded with alterations in RNA expressions in the kidney organoids. Transcriptomic profiling revealed significant differences in gene expression between syngeneic and allogeneic conditions, particularly highlighting the enrichment of the NF-κB and TNFα signaling pathways.. These pathways are known to play pivotal roles in mediating immune and inflammatory responses during renal allograft rejection [45, 46]. The NF-κB pathway is a central regulator of immune activation, promoting transcription of pro-inflammatory cytokines and adhesion molecules, while TNFα signaling contributes to both inflammation and apoptosis [47, 48]. Therefore, sustained activation of these pathways has been implicated in acute rejection and chronic allograft injury. To experimentally validate the RNA-seq findings, we performed qRT-PCR analysis of representative genes from these pathways. Notably, we observed upregulation of NF-κB and TNFα, along with increased expression of the downstream proinflammatory cytokine IL-6, and decreased expression of IκBα, a key negative regulator of NF-κB signaling [49, 50]. These results confirm that the activation of NF-κB and TNFα signaling in the kidney organoids is not only computationally inferred but also experimentally supported.

Although this study has yielded some significant results, it still has several limitations. First, although the co-culture system with kidney organoids and PBMCs provides a controlled environment, it does not capture the complexity of a full organ transplant scenario, which involves additional immune cells, vascular components, and systemic factors found in a living organism. Especially, it is important to consider the developmental state of the kidney organoids used in this study. We utilized organoids differentiated for 21 days, which consistently exhibit nephron-like structures and express key renal markers, enabling interaction with immune cells. However, their immature status may affect the allogeneic immune response by altering antigen presentation or cytokine profiles. Future studies should investigate how organoid maturation influences immune activation in transplant models. Second, tacrolimus was selected as the representative immunosuppressive agent due to its widespread and predominant use in contemporary renal transplantation protocols [42]. Nonetheless, further studies investigating the immunological impact of commonly used combination regimens, such as Tacrolimus with mycophenolate mofetil and corticosteroids, will be essential to more fully understand the in vivo relevance of our findings. Third, the focus on T cells and their subsets does not include other critical immune cells involved in transplant rejection, such as B cells, macrophages, and dendritic cells. The limited number of immune cells retrievable in vitro restricted the reliability of analysis for those cells. Detailed characterization of those cells would require a larger-scale experimental system. Future investigations using optimized models or in vivo approaches will be necessary to elucidate the comprehensive immune landscape during renal allograft rejection. Lastly, the generation of kidney organoids from hiPSCs and their subsequent use in co-culture systems require specialized skills and resources. Variability in organoid differentiation and co-culture conditions could impact the reproducibility and scalability of these findings for broader applications. A sophisticated protocol for creating kidney organoids that allows for long-term culture, along with the integration of a microfluidic system to incorporate vascular systems and secondary lymphoid tissues, could address these limitations.

## Conclusion

In conclusion, our study offers a comprehensive characterization of allogeneic interactions within the kidney organoid co-culture system, providing valuable insights into the pathophysiology of transplant rejection. By identifying key molecular and cellular changes, we establish a foundation for the development of targeted therapeutic interventions that aim to improve transplant outcomes and extend graft survival.

## Supplementary Information

Below is the link to the electronic supplementary material.

Supplementary file1 (DOCX 1966 KB)

Supplementary file2 (DOCX 17 KB)

## Data Availability

The data that support the findings of this study are available on request from the corresponding author. The data are not publicly available due to privacy or ethical restrictions.
