# Supplementary material for: Modeling of kidney allograft rejection using hiPSC-derived kidney organoids and HLA-mismatched PBMCs: an in vitro co-culture system
Source: Cell Mol Life Sci. 2025 Sep 2;82(1):333. doi: 10.1007/s00018-025-05867-7 (PMC12405138; doi:10.1007/s00018-025-05867-7)
Supplement: Supplementary file 1 — Supplementary file1 (DOCX 1966 KB) [file 18_2025_5867_MOESM1_ESM.docx]

**Table S1.** HLA typing results of healthy human subjects.

| **HLA typing** | **WTC-11 hiPSC** | | **HC hiPSC** | | **No. of mismatch allele** |
| --- | --- | --- | --- | --- | --- |
|  | Allele | Allele | Allele | Allele |  |
| HLA-A* | **26**:01:01:01 | **31**:01:02:01 | **26**:01:01:01 | **26**:02:01 | 1/4 |
| HLA-B* | **35**:01:01:01 | **51**:01:01:01 | **15**:01:01:01 | **27**:05:02:01 | 4/4 |
| HLA-C* | **03**:03:01:01 | **14**:02:01:01 | **02**:02:01 | **04**:01:01:01 | 4/4 |
| HLA-DRB1* | **04**:10:01 | **12**:01:01:01 | **12**:02:01:01 | **15**:01:01:01 | 2/4 |
| HLA-DQB1* | **03**:01:01:01 | **04**:02:01:01 | **03**:01:01:01 | **06**:02:01:01 | 4/4 |

**Table S2.** List of antibodies used for FACS detection.

| **Antibody** | **Format** | **Clone** | **Cat. no.** | **Company** |
| --- | --- | --- | --- | --- |
| HLA-ABC | PE-Cyanine7 | W6/32 | 25-9983-42 | Invitrogen, Camarillo, CA, USA |
| HLA-DR | APC-eFluor 780 | LN3 | 47-9956-42 | Invitrogen, Camarillo, CA, USA |
| PODXL | PE | B34D1.3 | 12-8873-42 | Invitrogen, Camarillo, CA, USA |
| LTL | Fluorescein | - | FL-1321-2 | Vector Laboratories, Burlingame, CA, USA |
| ECAD | APC | IgG2B | FAB18381A | R&D Systems, Minneapolis, MN, USA |
| CD3 | Super Bright 436 | UCHT1 | 62-0038-42 | Invitrogen, Camarillo, CA, USA |
| CD4 | FITC | RPA-T4 | 300505 | BioLegend, San Diego, CA, USA |
| CD8 | APC-eFluor 780 | RPA-T8 | 47-0088-42 | Invitrogen, Camarillo, CA, USA |
| CD45RO | PerCP-eFluor 710 | UCHL1 | 46-0457-42 | Invitrogen, Camarillo, CA, USA |
| CCR7 | APC | 3D12 | 17-1979-41 | Invitrogen, Camarillo, CA, USA |
| CD24 | BV421 | ML5 | 562789 | BD Horizon, San Jose, CA, USA |
| TIM-3 | PerCP-Cy5.5 | 7D3 | 567123 | BD Pharmingen, San Jose, CA, USA |
| NANOG | Unconjugated | 1E6C4 | sc-293121 | Santa Cruz, Santa Cruz, CA, USA |
| SSEA-4 | Unconjugated | 813-70 | sc-21704 | Santa Cruz, Santa Cruz, CA, USA |
| TRA-1-81 | Unconjugated | TRA-1-80 | sc-21706 | Santa Cruz, Santa Cruz, CA, USA |

**Table S3.** List of primary and secondary antibodies used for Immunofluorescence.

| **Antibody** | **Source** | **Dilution** | **Cat. no.** | **Company** |
| --- | --- | --- | --- | --- |
| HLA-ABC | Rabbit | 1/10 | IM0107 | Beckman Coulter, France |
| HLA-DR | Mouse | 1/30 | sc-33718 | Santa Cruz, Santa Cruz, CA, USA |
| PODXL | Goat | 1/500 | AF1658 | R&D Systems, Minneapolis, MN, USA |
| PODXL, Biotinylated | Goat | 1/100 | BAF1556 | R&D Systems, Minneapolis, MN, USA |
| LTL, Biotinylated |  | 1/100 | B-1325-2 | Vector Laboratories, Burlingame, CA, USA |
| ECAD | Rat | 1/50 | AB11512 | Abcam, Cambridge, UK |
| ECAD | Mouse | 1/100 | 610181 | BD Biosciences, San Jose, CA, USA |
| CD24 | Mouse | 1/50 | sc-19585 | Santa Cruz, Santa Cruz, CA, USA |
| TIM-3 | Goat | 1/50 | AF2365 | R&D System, Minneapolis, MN, USA |
| NANOG | Mouse | 1/100 | sc-293121 | Santa Cruz, Santa Cruz, CA, USA |
| SSEA-4 | Mouse | 1/100 | sc-21704 | Santa Cruz, Santa Cruz, CA, USA |
| TRA-1-81 | Mouse | 1/100 | sc-21706 | Santa Cruz, Santa Cruz, CA, USA |
| PAX6 | Mouse | 1/10 | sc-81649 | Santa Cruz, Santa Cruz, CA, USA |
| SM22A | Mouse | 1/250 | sc-53932 | Santa Cruz, Santa Cruz, CA, USA |
| FOXA2 | Mouse | 1/250 | sc-374376 | Santa Cruz, Santa Cruz, CA, USA |
| Anti-rabbit IgG-Alexa Fluor 488 | Donkey | 1/300 | A21206 | Invitrogen, Camarillo, CA, USA |
| Anti-mouse IgG-Alexa Fluor 488 | Donkey | 1/300 | A21202 | Invitrogen, Camarillo, CA, USA |
| Anti-goat IgG-Cyanine Cy3 | Donkey | 1/1000 | 705-166-147 | Jackson ImmunoResearch, West Grove, PA, USA |
| Streptavidin-Cyanine Cy3 |  | 1/1000 | 016-160-084 | Jackson ImmunoResearch, West Grove, PA, USA |
| Anti-mouse IgG-Alexa Fluor 647 | Donkey | 1/500 | A31571 | Invitrogen, Camarillo, CA, USA |
| Anti-rat IgG-Alexa Fluor 647 | Donkey | 1/500 | A78947 | Invitrogen, Camarillo, CA, USA |
| Anti-goat IgG-Alexa Fluor 647 | Donkey | 1/500 | A21447 | Invitrogen, Camarillo, CA, USA |

**Table S4.** List of sequences of primers used for qRT-PCR.

| Target name | Direction | Primer sequence (5’-3’) | Size (bp) |
| --- | --- | --- | --- |
| NFκB | Forward | GAGACATCCTTCCGCAAACT | 101 |
|  | Reverse | ACTGGTCAGAGACTCGGTAAA |  |
| IκBα | Forward | CTCCGAGACTTTCGAGGAAATAC | 134 |
|  | Reverse | GGTCCTTCCTGCCCATAATC |  |
| TNFα | Forward | CCAGGGACCTCTCTCTAATCA | 106 |
|  | Reverse | TCAGCTTGAGGGTTTGCTAC |  |
| IL-6 | Forward | CCAGGAGAAGATTCCAAAGATGTA | 94 |
|  | Reverse | CGTCGAGGATGTACCGAATTT |  |
| GAPDH | Forward | GACCAGGCAATCATGGAGAA | 10 |
|  | Reverse | CTTCGGTGAAAGCCCTTAGT |  |

**
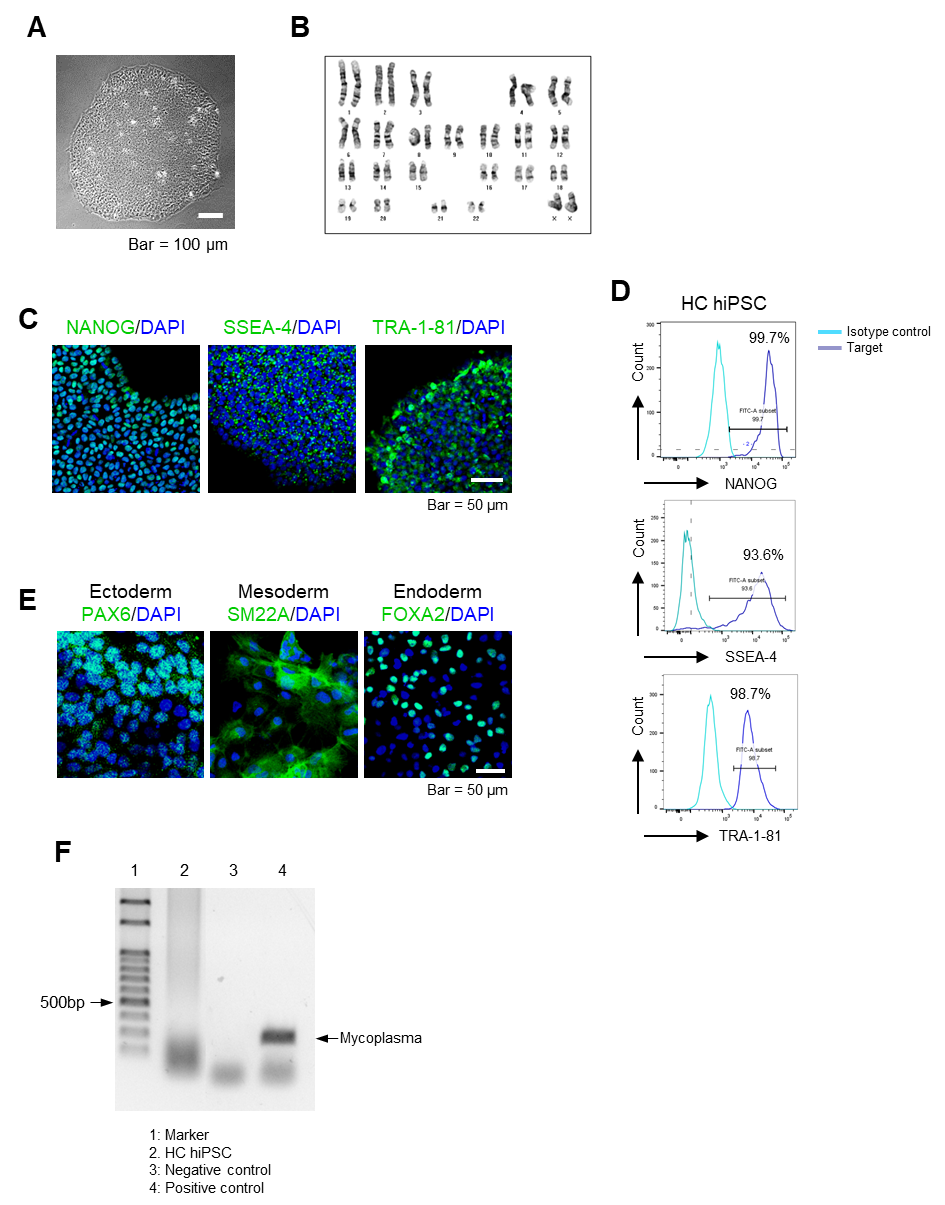
**

**Figure S1** **Characterization of healthy control (HC) hiPSC line.** (**A**) Morphology of HC hiPSCs. Scale bar = 100 μm. (**B**) Chromosome karyotyping of HC hiPSCs. (**C**) Representative immunofluorescence images of pluripotency markers NANOG, SSEA-4, and TRA-1-81. Scale bar = 50 μm. (**D**) Flow cytometry analysis of cells expressing NANOG, SSEA-4, and TRA-1-81. (**E**) Representative immunofluorescence images of three germ layer markers. Ectoderm, mesoderm, and endoderm differentiation were detected by PAX6, SM22A, and FOXA2A, respectively. Scale bar = 50 μm. (**F**) Mycoplasma detection by PCR, negative.

**
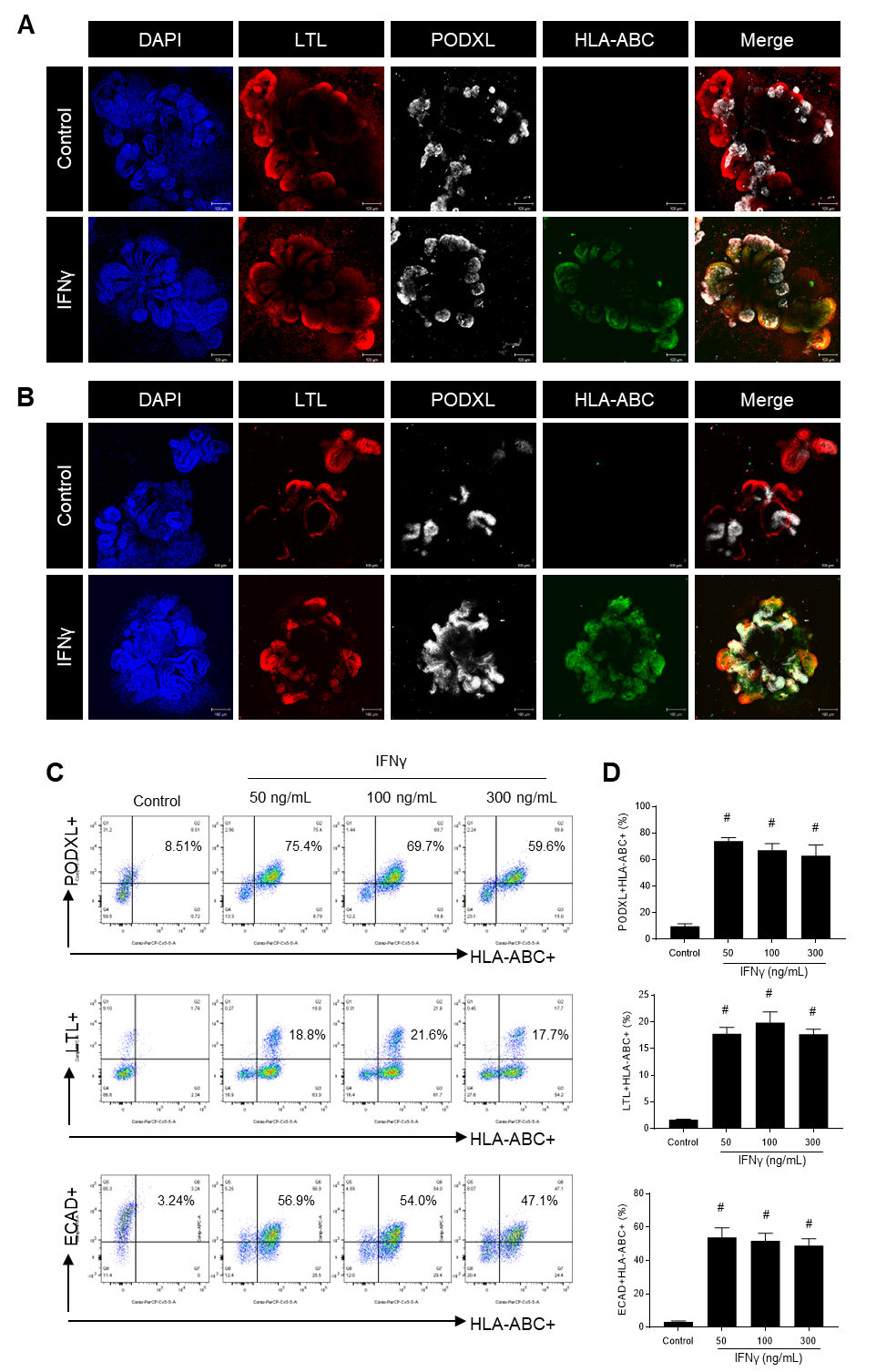
**

**Figure S2** **Effect of IFNγ treatment on the expression of HLA-ABC in kidney organoids.** Kidney organoids derived WT hiPSC were treated with 50~300 ng/mL of recombinant human IFNγ protein for 24 h. Representative immunofluorescence images (**A** and **B**), flow cytometric dot plots (**C**) and its quantitative graphs (**D**) of HLA-ABC in the nephron markers, PODXL, LTL, and ECAD in the IFNγ treated kidney organoids. Scale bar = 100 μm PODXL, podocalyxin; LTL, lotus tetragonolobus lectin; ECAD, e-cadherin. Data are presented as mean ± SE. ^#^*P* < 0.05 vs. syngeneic group.

**
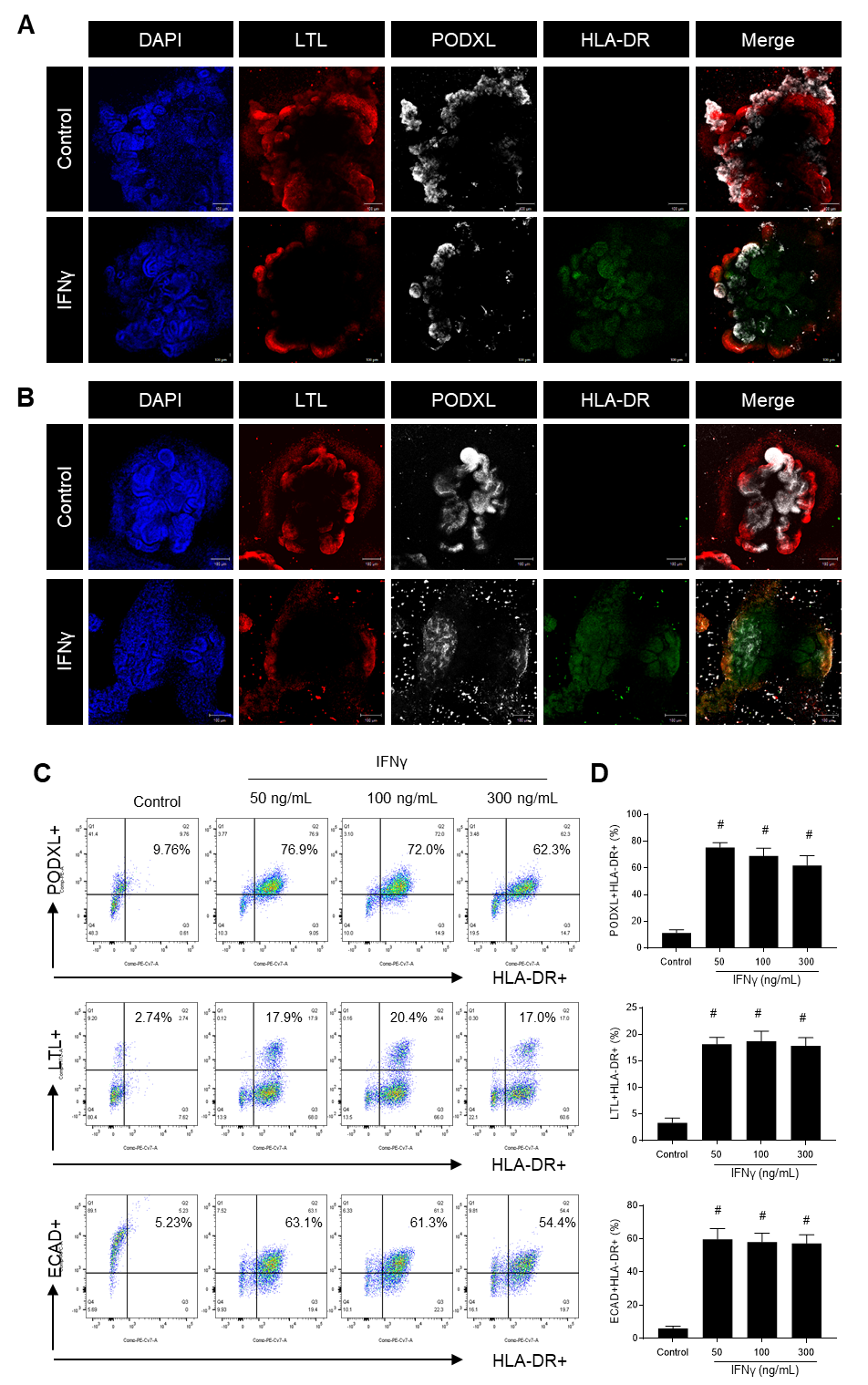
**

**Figure S3** **Effect of IFNγ treatment on the expression of HLA-DR in kidney organoids.** Kidney organoids derived WT hiPSC were treated with 50~300 ng/mL of recombinant human IFNγ protein for 24 h. Representative immunofluorescence images (**A** and **B**), flow cytometric dot plots (**C**) and its quantitative graphs (**D**) of HLA-DR in the nephron markers, PODXL, LTL, and ECAD in the IFNγ treated kidney organoids. Scale bar = 100 μm PODXL, podocalyxin; LTL, lotus tetragonolobus lectin; ECAD, e-cadherin. Data are presented as mean ± SE. ^#^*P* < 0.05 vs. syngeneic group.

**
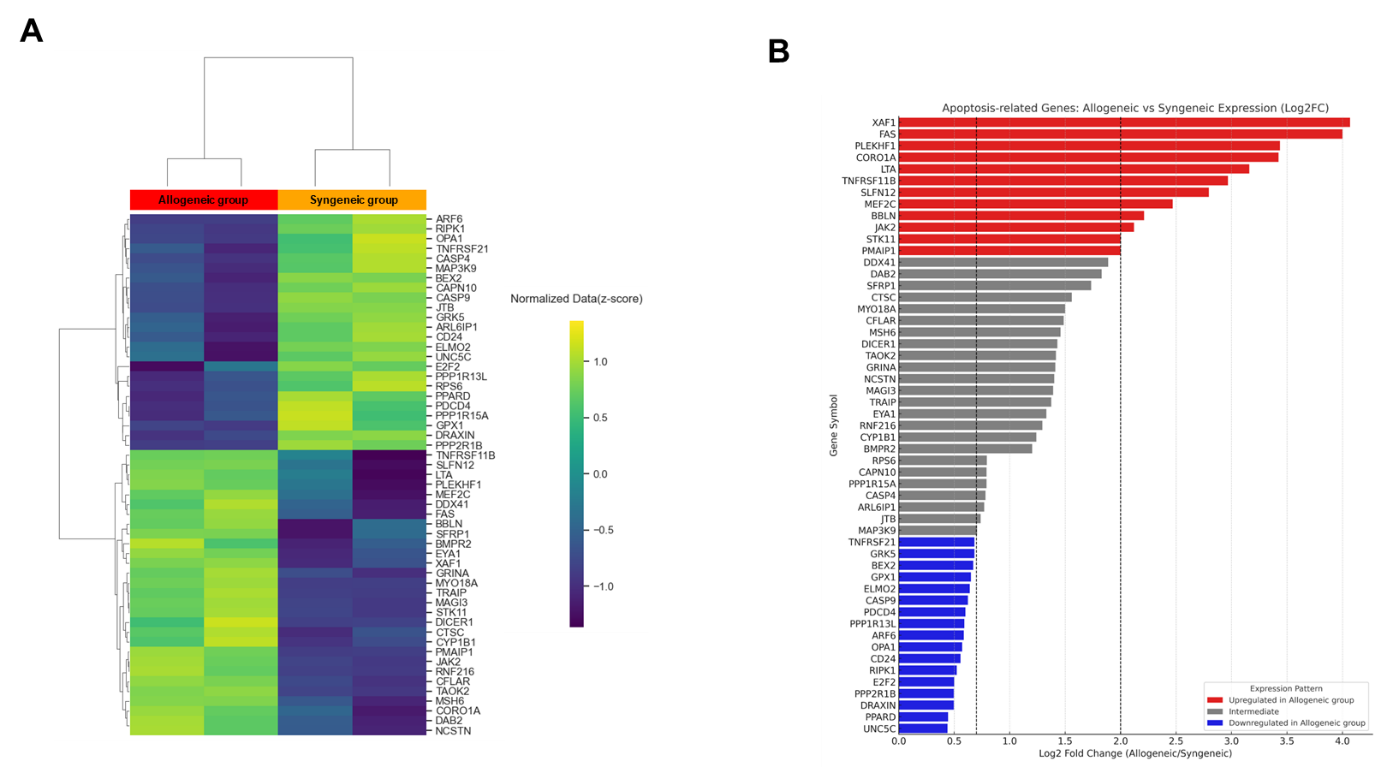
**

**Figure S4. Differential expression of apoptosis-related genes in allogeneic vs. syngeneic groups. (A)** Heatmap showing the expression patterns of apoptosis-related genes in allogeneic and syngeneic groups. Each row represents a gene, and each column represents an individual sample. Expression values are scaled using Z-score normalization across genes. Samples from the allogeneic group (red bar) and syngeneic group (orange bar) are clearly separated by hierarchical clustering, and multiple apoptosis-related genes exhibit distinct expression patterns between the groups. **(B)** Bar plot of the log2 fold change (log2FC) of apoptosis-related genes comparing the allogeneic group to the syngeneic group. Genes with log2FC ≥ 2 are classified as “upregulated in the allogeneic group” (red), whereas those with log2FC ≤ 0.7 are categorized as “downregulated in the allogeneic group” (blue). Intermediate genes are shown in gray. Selected pro-apoptotic genes, including *XAF1*, *FAS*, and *PMAIP1*, were significantly upregulated in the allogeneic group, while several anti-apoptotic or survival-related genes such as *CD24*, *RIPK1*, and *PPP1R13L* were downregulated.
