# Supplementary material for: Modeling of kidney allograft rejection using hiPSC-derived kidney organoids and HLA-mismatched PBMCs: an in vitro co-culture system
Source: Cell Mol Life Sci. 2025 Sep 2;82(1):333. doi: 10.1007/s00018-025-05867-7 (PMC12405138; doi:10.1007/s00018-025-05867-7)
Supplement: Supplementary file 2 — Supplementary file2 (DOCX 17 KB) [file 18_2025_5867_MOESM2_ESM.docx]

**Materials and Methods for supplemental figures**

**iPSC differentiation**

Healthy control, HC iPSCs were generated using PBMCs, as previously described (Kim Y et al., 2016). Briefly, PBMCs were cultured for 4 days at 37°C in an incubator with 5% CO_2_ in StemSpan medium (09650; STEMCELL Technologies, Vancouver, Canada), which includes StemSpan CC100 (02690; STEMCELL Technologies), to expand CD34-positive cells. The expanded PBMCs were transfected using the CytoTune-iPS Sendai Reprogramming Kit (A16517; Life Technologies, Carlsbad, CA, USA), which includes the Yamanaka factors (Oct4, Sox2, KLF4, and c-Myc). PBMCs were induced to form iPSCs via centrifugation; the resultant attached cells were expanded and purified by colony picking.

**Kidney organoid differentiation from hiPSCs and IFNγ treatment**

Wild type (WT) and HC hiPSCs were differentiated into kidney organoids following the previously published protocol (Freedman et al., 2015; Cruz et al., 2017; Lie et al., 2020). Briefly, hiPSCs were plated in mTeSR1 medium (05850; STEMCELL Technologies Vancouver, Canada) supplemented with 10 μM Y-27632 (1293823; Biogems, Westlake Village, CA, USA) onto 24-well plates pre-coated with 1.25% Corning Matrigel® hESC Qualified Matrix. After 24 hours, the medium was exchanged with 2.5% of Matrigel® in mTeSR1. On the fourth day, the medium was replaced with Advanced RPMI (1263302; Thermo Fisher Scientific, Grand Island, NY, USA) supplemented with 12 µM CHIR-99021 (STEMCELL Technologies). Approximately 36 hours later, the medium was changed to Advanced RPMI with B27 supplement (17504044, Thermo Fisher Scientific). Organoids were cultured in this medium until collection on day 21. On day 20, IFNr at the final concentration of 50 ~ 300 ng/mL were treated in the kidney organoids for 24 h. Next day, kidney organoids in each well were removed for further experiments.

**Tri-lineage differentiation**

Regarding trilineage differentiation, a StemMACS™ Trilineage Differentiation Kit (Miltenyi Biotec, Gaithersburg, MD, USA) was used. Putative hiPSCs were cultivated for seven days in three different chemically defined media, driving the differentiation into three germ layers. The three differentiated germ layers were fixed in 4% paraformaldehyde and stained with the following antibodies: anti-PAX6 antibody for the ectoderm, anti-SM22A antibody for the mesoderm, and anti-FOXA2 antibody for the endoderm.

**Karyotype analysis**

Pluripotent cells were cultured in culture plates coated with Matrigel in conditioned media for 3 ~ 5 days. The cells were transported to GenDix Research Center (GenDix Inc., Seoul, Republic of Korea), where cell harvest and karyotype analysis of metaphase chromosomes was performed using G-banding.

**Mycoplasma detection**

Mycoplasma analysis was performed using the e-Myco VALiD Mycoplasma PCR kit (iNtRON Biotechnology) and was absent in all cases.

**Immunofluorescence**

hiPSCs or kidney organoids were washed once with phosphate-buffered saline (PBS), fixed with 4% paraformaldehyde for 10 min at 4°C, and were blocked in 5% donkey serum in PBS-T (0.3% Triton X-100 in PBS) for 1 h at room temperature (RT). hiPSCs or kidney organoids were incubated with primary antibodies listed in the Table S2 for overnight at 4°C. Then, the cells were stained with secondary antibodies, Alexa Fluor 488-donkey anti-mouse IgG (A32766, 1:250; Invitrogen, Camarillo, CA, USA), Alexa Fluor 647-donkey anti-goat IgG (A32849, 1:250; Invitrogen), Cyanine3(Cy3)-streptavidin (016-160-084, 1:1000; Jackson ImmunoResearch, West Grove, PA, USA), Alexa Fluor 488-donkey anti-rat IgG (A48269, 1:250; Invitrogen), and Cy3-conjugated donkey anti-rabbit (711-165-152, 1:1000; Jackson ImmunoResearch). Nucleic acid staining was performed by incubation with 4′,6-diamidine-2-phenylindole (10236276001, DAPI, 1:5000; Roche, Basel, Switzerland) for 30 min at room temperature. Images were obtained using a Zeiss LSM700 confocal microscope (Carl Zeiss MicroImaging GmbH, Jena, Germany).

**Flow cytometry**

hiPSCs cells were dissociated using TE (15400054; Life Technologies). The cells were washed twice with FACS buffer (phosphate-buffered saline [PBS] containing 1% bovine serum albumin and 10 mM sodium azide), permeabilized for 30 min using flow cytometry fixation and permeabilization solution (554714; BD Biosciences, San Jose, CA, USA), washed with wash buffer, and stained with antibodies listed in the Table S2. Cells were analyzed using a FACS Canto II flow cytometer (BD Biosciences). The data were analyzed using FlowJo™ Software v10.10 (Becton, Dickinson & Company, Ashland, OR, USA).
